# Feasibility, Accuracy, and Repeatability of Suprathreshold Saccadic Vector Optokinetic Perimetry

**DOI:** 10.1167/tvst.5.4.15

**Published:** 2016-08-31

**Authors:** Ian C. Murray, Lorraine A. Cameron, Alice D. McTrusty, Antonios Perperidis, Harry M. Brash, Brian W. Fleck, Robert A. Minns

**Affiliations:** 1 University of Edinburgh, Edinburgh, Scotland, UK; 2 Glasgow Caledonian University, Glasgow, Scotland, UK; 3 Heriot Watt University, Edinburgh, Scotland, UK; 4 Princess Alexandra Eye Pavilion, Edinburgh, Scotland, UK; 5 Royal Hospital for Sick Children, Edinburgh, Scotland, UK

**Keywords:** perimetry, visual fields, SVOP, saccadic eye movements

## Abstract

**Purpose:**

To evaluate feasibility, accuracy, and repeatability of suprathreshold Saccadic Vector Optokinetic Perimetry (SVOP) by comparison with Humphrey Field Analyzer (HFA) perimetry.

**Methods:**

The subjects included children with suspected field defects (*n* = 10, age 5–15 years), adults with field defects (*n* = 33, age 39–78 years), healthy children (*n* = 12, age 6–14 years), and healthy adults (*n* = 30, age 16–61 years). The test protocol comprised repeat suprathreshold SVOP and HFA testing with the C-40 test pattern. Feasibility was assessed by protocol completeness. Sensitivity, specificity, and accuracy of SVOP was established by comparison with reliable HFA tests in two ways: (1) visual field pattern results (normal/abnormal), and (2) individual test point outcomes (seen/unseen). Repeatability of each test type was assessed using Cohen's kappa coefficient.

**Results:**

Of subjects, 82% completed a full protocol. Poor reliability of HFA testing in child patients limited the robustness of comparisons in this group. Sensitivity, specificity, and accuracy across all groups when analyzing the visual field pattern results was 90.9%, 88.5%, and 89.0%, respectively, and was 69.1%, 96.9%, and 95.0%, respectively, when analyzing the individual test points. Cohen's kappa coefficient for repeatability of SVOP and HFA was excellent (0.87 and 0.88, respectively) when assessing visual field pattern results, and substantial (0.62 and 0.74, respectively) when assessing test point outcomes.

**Conclusions:**

SVOP was accurate in this group of adults. Further studies are required to assess SVOP in child patient groups.

**Translational Relevance:**

SVOP technology is still in its infancy but is used in a number of centers. It will undergo iterative improvements and this study provides a benchmark for future iterations.

## Introduction

Identifying visual field defects is crucial to aid the diagnosis and management of many ocular and neurological diseases. In children, knowledge of visual field defects is especially important in patients with cerebral visual impairment,^1,2^ visual pathway tumors,^3,4^ pediatric glaucoma,^5^ and patients taking certain medication (e.g., Vigabatrin).^6,7^ Despite the clinical need, the difficulties associated with performing Standard Automated Perimetry (SAP) in children are well documented.^8–11^ Manual kinetic perimetry is a popular technique used with children between the ages of 5 and 9 years because the test can be tailored to the child's ability.^12–14^ However, the technique still requires the child's cooperation and understanding, and results can be dependent upon the examiner's testing skills.^15^ Currently, visual field assessment in children under the age of 5 years is typically limited to the assessment technique of confrontation, which uses a child's natural eye movement responses but is qualitative and imprecise.^16^ The use of algorithms designed to provide faster testing time for SAP such as Swedish Interactive Threshold Algorithm (SITA)^17,18^ and tendency-oriented perimetry (TOP)^19^ have been assessed with child subjects. However, the youngest aged child capable of producing reliable results remains approximately 7 to 8 years.^20–22^ These studies do not address the inherent difficulties children have with traditional perimetry techniques. Namely, (1) continuous central fixation, (2) understanding of the complex test method sequences (field-point recognition and response), and (3) the use of a head and chin rest. In addition, algorithms such as SITA are designed specifically for adult populations.^23^

A number of recent studies have specifically aimed to address the lack of suitable perimetry methods for children, namely (1) manual visual field testing approaches, such as the behavioral visual field test has proven reliable in young or neurologically impaired patients,^24^ (2) perimetry testing that encourages interaction by using a computer game format makes the task engaging for children,^25^ (3) KidzEyes, a modern form of video-assisted preferential looking perimetry where the examiner views the natural eye gaze response to peripheral stimuli.^26^ These recent approaches all still require a form of central fixation by the patient and either use nonstandard peripheral stimuli or are partially subjective on the part of the examiners.

The development of a more patient friendly form of perimetry may also be of benefit to adults. Data from qualitative studies indicate that patients find visual field testing more laborious and demanding than other vision tests^27^ and patients have reported that they would prefer a more modernized visual field test to improve their experience.^28^ A more natural test that is easier to perform could be a useful adjunct for assessing the visual field in adult patients unable to cope with the demands of SAP.

Saccadic vector optokinetic perimetry (SVOP) is a technique originally developed to enable visual field assessment in children unable to perform conventional forms of perimetry.^29–32^ SVOP was designed to combat the problems children have with conventional techniques by combining multifixation perimetry,^33^ and eye tracking technology to automatically assess eye movement responses to visual field stimuli. This study aims to evaluate the feasibility, accuracy, and repeatability of suprathreshold SVOP by comparison with equivalent tests using the Humphrey Field Analyser (HFA).

## Subjects and Methods

### Subjects

The study adhered to the tenets of the Declaration of Helsinki and was approved by the South East Scotland Research Ethics Committee, NHS Lothian. Informed consent was obtained from all participants and their parent or guardian as required.

An unselected nonconsecutive series of children and adult patients in Edinburgh attending the ophthalmology clinic at the Royal Hospital for Sick Children or visual field assessment clinics at the Princess Alexandra Eye Pavilion, respectively, were recruited. Included patients had known or suspected visual field defects. A similar number of healthy subjects were recruited who had no history of ophthalmological or neurological disease likely to cause a visual field defect. Children included in the study were aged 5 to 15 years and adults were 16 years or older. Subjects with severe eye movement disorders which might preclude accurate SVOP testing were excluded from the study, but cases with strabismus and/or nystagmus were included.

### Test Protocol

A complete test protocol comprised eight tests over two sessions on the same day. Tests in each session consisted of a right and a left eye suprathreshold visual field test on both devices (SVOP and HFA). The device used first was randomized in the first session and the order was reversed in the second session. Suitable breaks between tests were provided.

#### SVOP Tests

**Figure 1:**
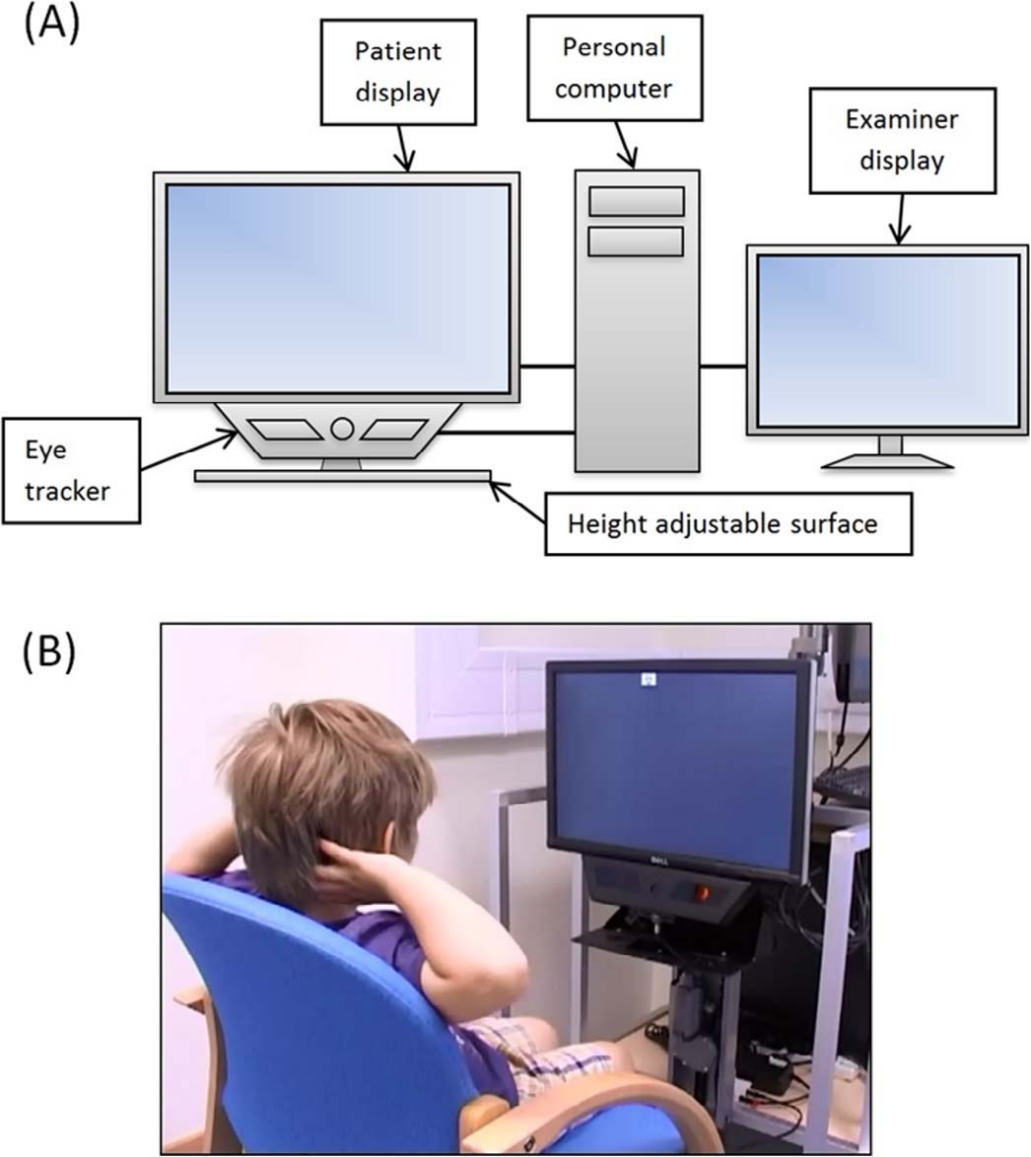
The SVOP system. (A) A schematic of the system components. (B) A child performing an SVOP test.

The SVOP system used was a research prototype device (Fig. 1). It comprised a personal computer (Dell Precision 380 workstation; Dell, Round Rock, TX), a 20″ patient display screen (Dell 2005FPW), and an eye tracker (X50; Tobii Technology, Stockholm, Sweden). The position of the patient display screen could be adjusted for different patient heights and a secondary display screen (out of sight from the patient) was used by the operator to input patient details, set-up tests, and monitor test progress. The only task required of the subject was to follow their natural reaction to visually fixate towards the area where visual field “test stimuli” were presented. A software algorithm automatically determined if subjects could perceive the test stimuli based on the direction and amplitude of a subject's eye gaze response.^29^ The eye tracker was noncontact and provides “real time” (sample rate of 50 Hz and typical latency of 25–35 ms) data on: (1) three-dimensional (3D) eye position relative to the eye tracker, and (2) the point of gaze on the display screen. This allows: (1) the screen coordinates of visual field stimuli to be calculated in “real time,” and (2) patient eye gaze responses to visual field stimuli to be automatically assessed in “real time.”

Subjects were positioned in front of the patient display screen using appropriate seating for their age. The screen was positioned such that the subject's eyes were located approximately centrally in front of the display screen and 55 to 60 cm away from the eye tracker's camera. This procedure was aided using an on-screen graphic, which provided a real-time representation of the location of the subject's eyes.

Prior to each SVOP test, a calibration procedure was performed in which subjects were required to follow a visual stimulus with their gaze to five different screen locations. This procedure allowed characteristics of the subject's eyes (such as pupil position and shape) to be determined and used together with a mathematical 3D eye model in order to produce accurate gaze position data for that subject. The calibration stimulus used was a circle with a central dot, which moved to each location in a random order.

The visual field test stimuli were all of size Goldmann III (0.43° angular diameter) and duration 200 ms. By using a calibrated patient display screen, stimuli of defined luminance levels were produced using a process, which has since been refined further.^31^ A luminance level equivalent to 14 dB on the HFA (stimulus and background luminance of 137 and 10 cd/m^2^, respectively) was used in this suprathreshold test. Participants were given the simple instruction “If you see anything flash up on the screen look at it or where you thought it flashed up.”

**Figure 2:**
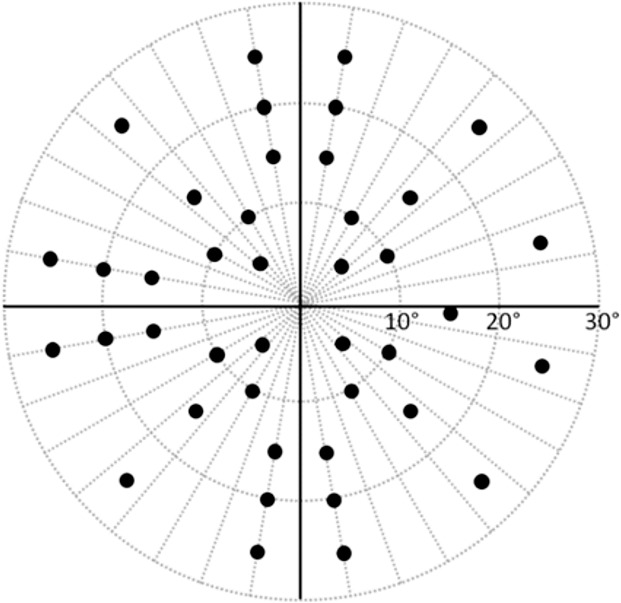
Right eye test pattern used for the HFA and SVOP tests. The left eye test pattern is the mirror image of the right eye pattern. The blind spot location is a fixed test point in SVOP tests.

A test pattern consisting of 41 test points (Fig. 2) was employed. This test pattern is equivalent to the HFA's C-40 screening test patterns with the addition of a test point located at the natural blind spot (positioned 15° temporally and 1.5° below the midline).

#### HFA Tests

The HFA C-40 screening (suprathreshold) test was used as the reference standard. The HFA's default screening test settings present visual field stimuli 6 dB brighter than the expected threshold value at each test location for the patient's age.^34^ The age-matched values are taken from a proprietary database of normative subject data. In order to replicate the SVOP test, the HFA test mode setting was changed to “single intensity” and set at a level of 14 dB. Visual field test stimuli used were of size Goldmann III (0.43° angular diameter) and duration 200 ms.

For the HFA test, subjects were positioned appropriately on the chin and forehead rest to align their test eye with the central fixation stimulus. Subjects were instructed to fixate centrally for the duration of the test and to respond with a button press when they saw a light flash up.

### Data Analysis

#### Feasibility of Protocol and Perimetry Tests

The feasibility of the testing protocol employed in the study was assessed by analyzing the extent of the protocol completed by the participants. The feasibility of each test method (SVOP and HFA) was assessed by analyzing the number of tests that were completed during the first test session. In addition, an analysis of the HFA reliability indices (false-positive, false-negative, and fixation loss rates) was performed to identify unreliable HFA test results. False-positive responses were those when the subject responded even when no stimulus was presented, false-negative responses were those when the subject didn't respond to a stimulus that was previously seen, and fixation losses were responses to stimuli shown in the blind spot location. Unreliable HFA test results were those with a false-positive response rate exceeding 15%, as recommended by manufacturer guidelines.^35^ Moreover, in order to capture further potentially unreliable tests, those with false-negative responses greater than 33% or fixation losses greater than 33% were also categorized as unreliable and excluded from all subsequent analysis. SVOP tests did not have reliability indices similar to the HFA because SVOP only presents a stimulus once fixation has been achieved, and the response mechanism for stimuli to been classified as seen requires a specific eye movement in the direction of the presented stimulus (rather than just a button press as is the case with the HFA). SVOP tests excluded from further analysis were those that were less than 95% complete.

#### Accuracy of SVOP by Comparison with HFA

Sensitivity, specificity, and accuracy of the SVOP test was calculated using the test results from the first session and using HFA test results as the reference standard. Sensitivity and specificity was assessed in two ways:

1. Analysis of the overall visual field pattern: the SVOP and HFA test results were classified as normal or abnormal by a panel of three masked graders (one ophthalmologist and two research optometrists). Abnormality was defined according to routine clinical practice as two or more contiguous unseen points, or three or more noncontiguous unseen points across the whole test result, excluding the blind spot location; and
2. Analysis of the visual field points: direct comparison of the individual visual field point results of “seen” or “unseen” from SVOP and HFA tests.

#### Repeatability of SVOP and HFA

Cohen's kappa coefficient was used to obtain a measure of agreement between repeated tests. The analysis was performed using the two approaches described in the previous section (analysis of the whole visual field plot and point by point analysis). The agreement according to the kappa statistic was interpreted as follows: slight, less than or equal to 0.20; fair, 0.21 to 0.40; moderate, 0.41 to 0.60; substantial, 0.61 to 0.80; and excellent, greater than 0.80.^36^

#### Test Times

SVOP and HFA test times from completed tests performed over the first test session were compared. Normality assumption was assessed by inspection of histograms and using Shapiro-Wilk tests. Student *t*-tests were used for group comparison of normally distributed variables and Wilcoxon rank-sum test for continuous nonnormal variables. All tests were two-sided and a *P* value less than 0.05 was considered statistically significant.

## Results

### Subjects

Eighty-five subjects participated in the study and were categorized into four subject groups (Table 1). The diagnoses of the adult and child patients are detailed in Table 2.

**Table 1:**
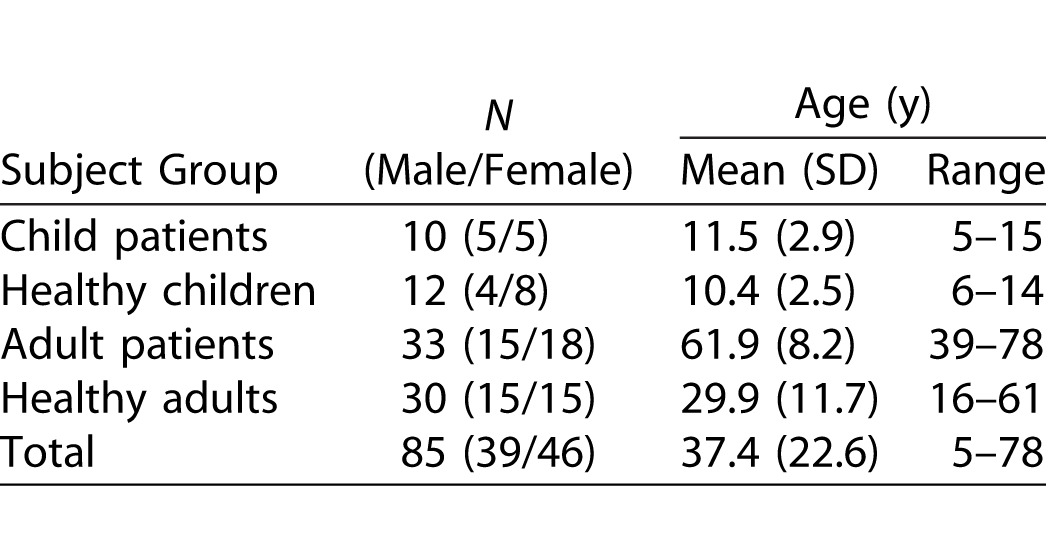
Subjects

**Table 2:**
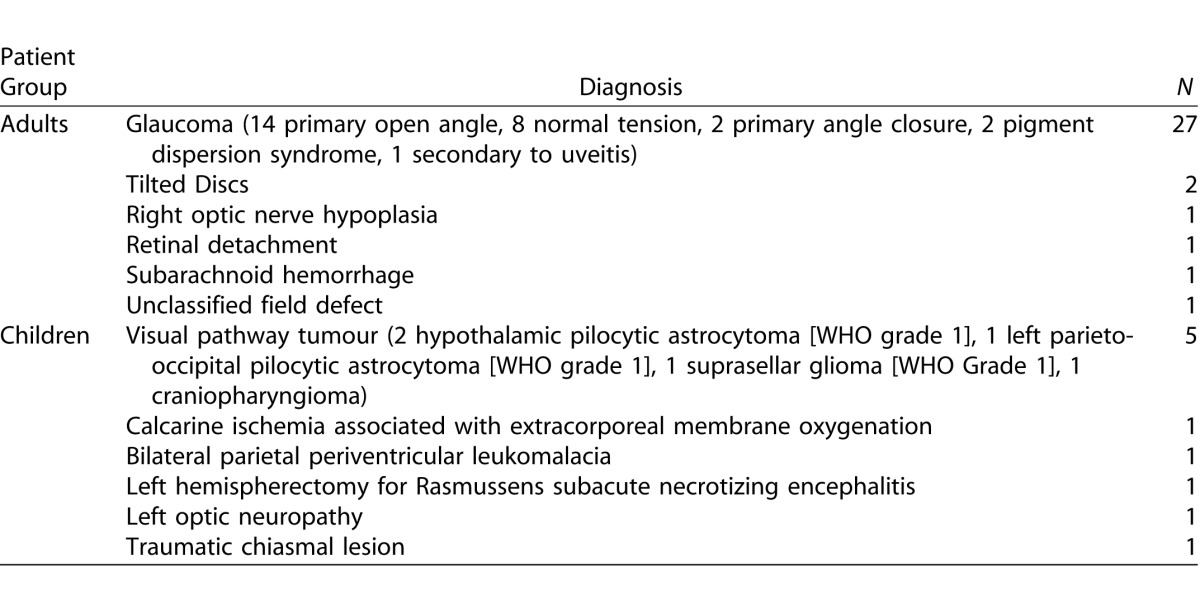
Patient Diagnoses

### Feasibility of Protocol and Perimetry Tests

Table 3 shows the extent of the test protocol performed by the participants in each subject group and also shows the number of SVOP and HFA tests performed and resulting comparison and repeatability pairs prior to any test exclusions. Of subjects, 82% completed a full protocol. There were several reasons for an incomplete protocol. Where only a single session was performed in the adult groups the reasons were (1) unable to continue due to time constraints (1 healthy adult and 1 adult patient), or (2) unwilling to continue due to fatigue after performing other visual field testing the same day (1 adult patient). In the child patient group, a decision not to continue to the second session was made in five patients. These decisions were made because of long testing times, poor performance on the HFA, or incomplete SVOP testing. Where the protocol was half performed due to only a single eye being tested, but over both sessions, the reason for this was either no vision in one eye (2 child patients) or inaccurate eye tracking for one eye which impeded SVOP testing (1 child patient and 1 adult patient). Where less than half the protocol was performed the reason was due to poor eye tracking for both the left and right SVOP tests (3 adult patients).

**Table 3:**
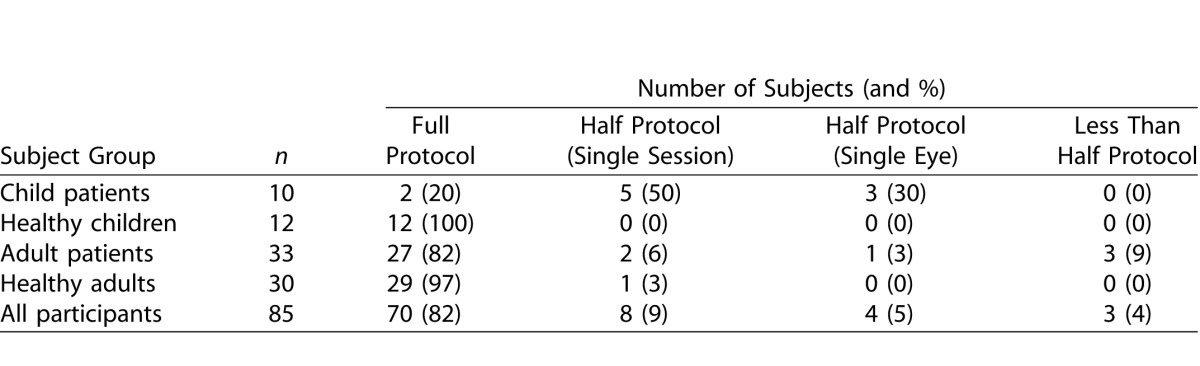
The Extent of the Test Protocol Performed, the Number of Tests Performed, and the Number of Test Comparison and Repeatability Pairs (Prior to Test Exclusions) in Each Subject Group

**Table 3:**
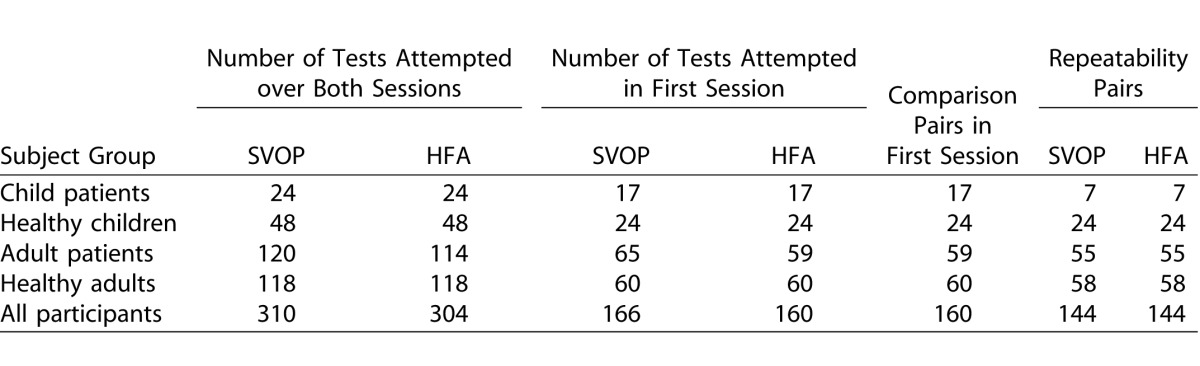
Extended

**Figure 3:**
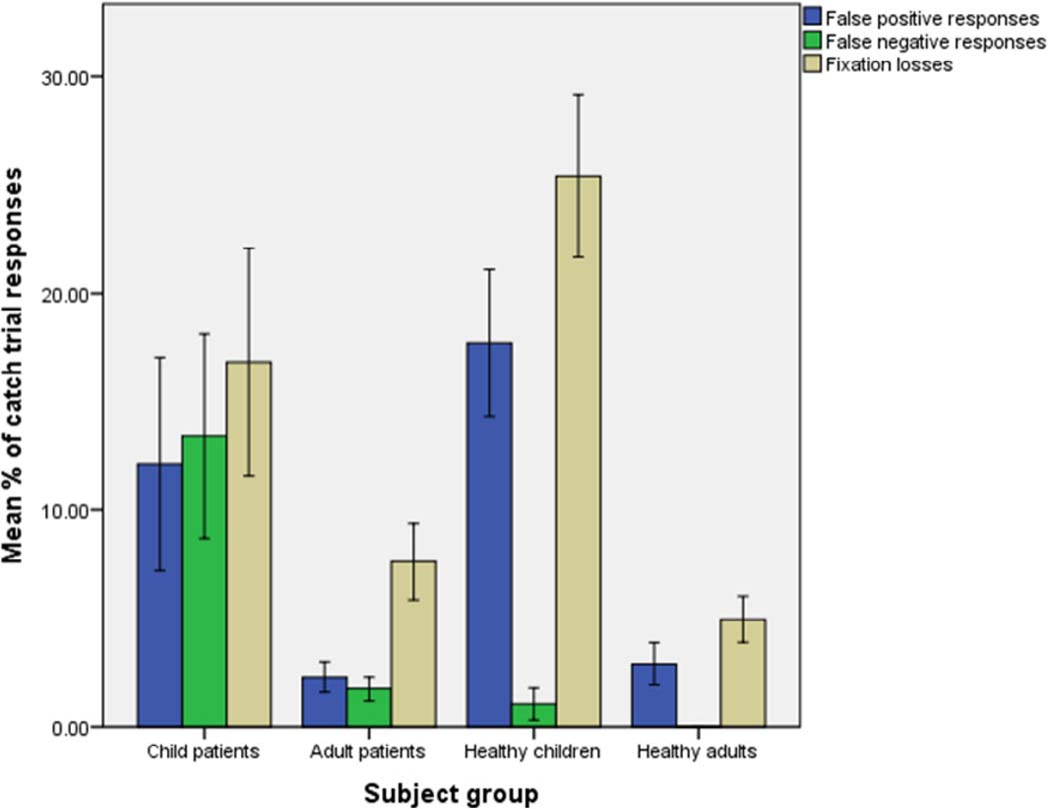
Humphrey Field Analyser reliability index responses. Data shown is the mean of the test reliability index rates (percentage false-positive responses, false-negative responses, and fixation losses) ±1 standard error of the mean.

Table 4 details the number of tests excluded from analysis and the resultant remaining test comparison pairs from the first test session, and test repeatability pairs used for further analysis. Additionally, Figure 3 shows the average reliability index rates for all HFA tests performed (average percentage of false-positive responses, false-negative responses, and fixation losses) for each subject group.

**Table 4:**
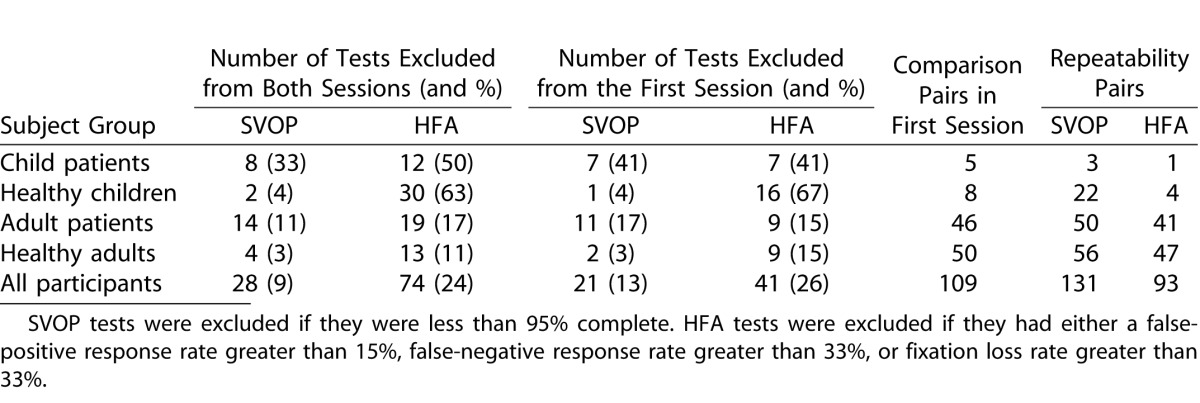
SVOP and HFA Tests Excluded from Analysis and Final Resultant Test Comparison and Repeatability Pairs

### Accuracy of SVOP by Comparison with HFA

#### Analysis of the Overall Visual Field Pattern

Tables 5, 6, and 7 show the contingency tables for the children, adults, and all subjects, respectively. Positive test results were those categorized as abnormal (defined in “data analysis” section). Also shown are the positive and negative predictive values, the prevalence of abnormal visual field results and the accuracy of SVOP.

**Table 5:**
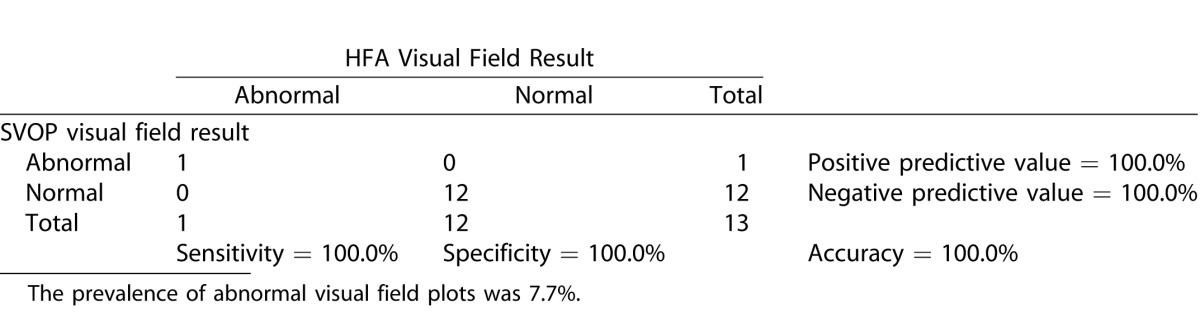
Contingency Table Comparing SVOP Test Results with HFA Test Results in the Child Groups

**Table 6:**
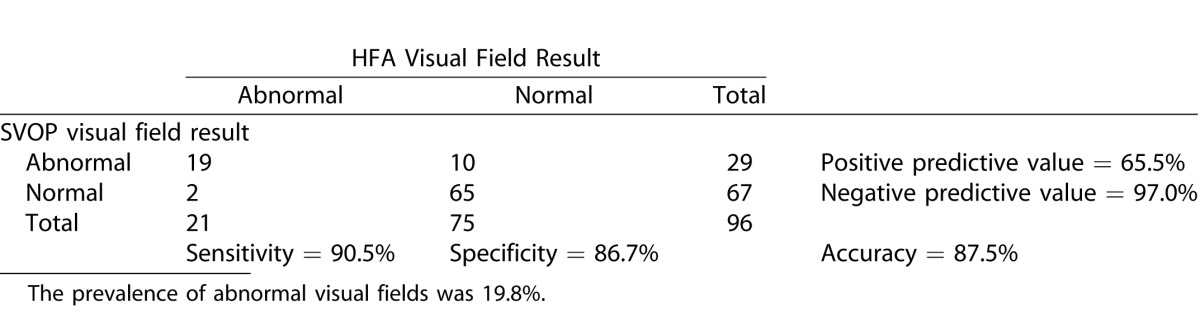
Contingency Table Comparing SVOP Test Results with HFA Test Results in the Adult Groups

**Table 7:**
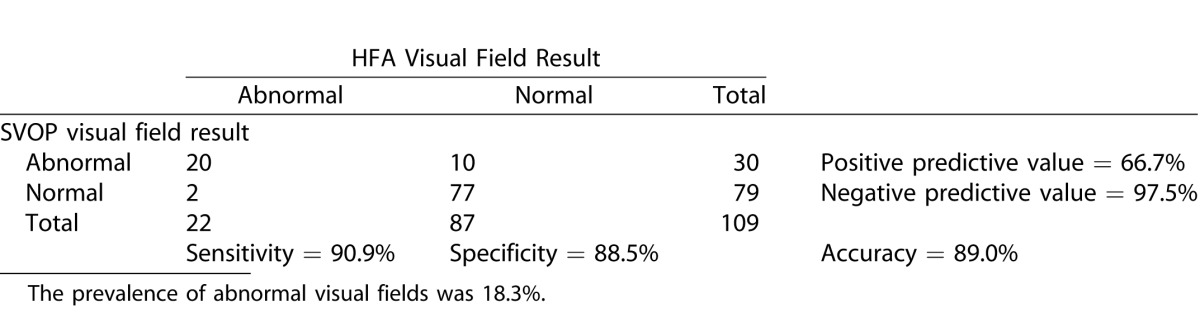
Contingency Table Comparing SVOP Test Results with HFA Test Results in all Groups

#### Analysis of the Visual Field Points

Sensitivity and specificity of SVOP was also calculated by comparing all the individual test points in the SVOP and HFA results. Tables 8, 9, and 10 show the contingency tables for the children, adults, and all subjects, respectively. Positive test points were those categorized as “unseen.”

**Table 8:**
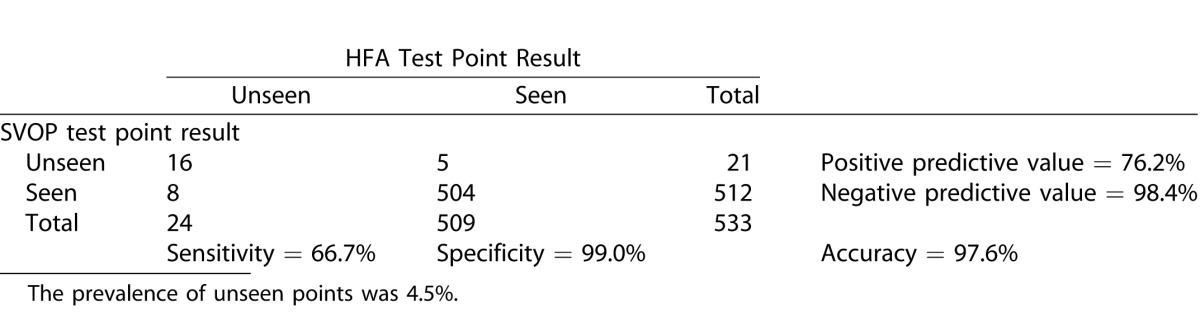
Contingency Table Comparing SVOP Test Point Results with HFA Test Point Results in the Child Groups

**Table 9:**
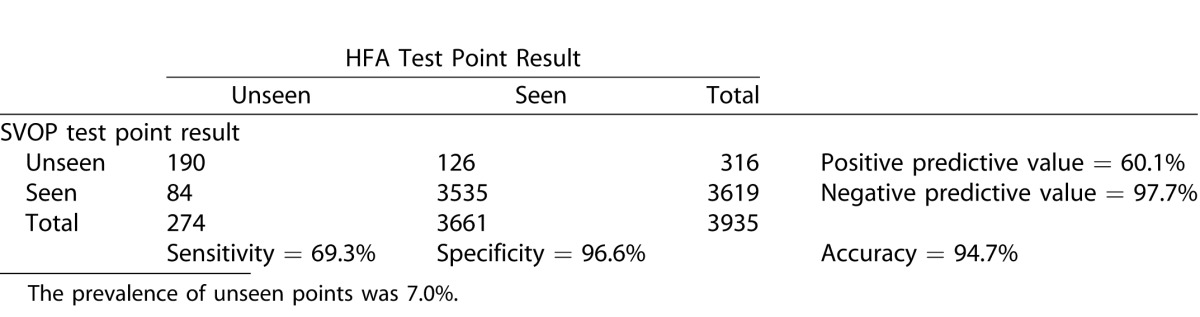
Contingency Table Comparing SVOP Test Point Results with HFA Test Point Results in the Adult Groups

**Table 10:**
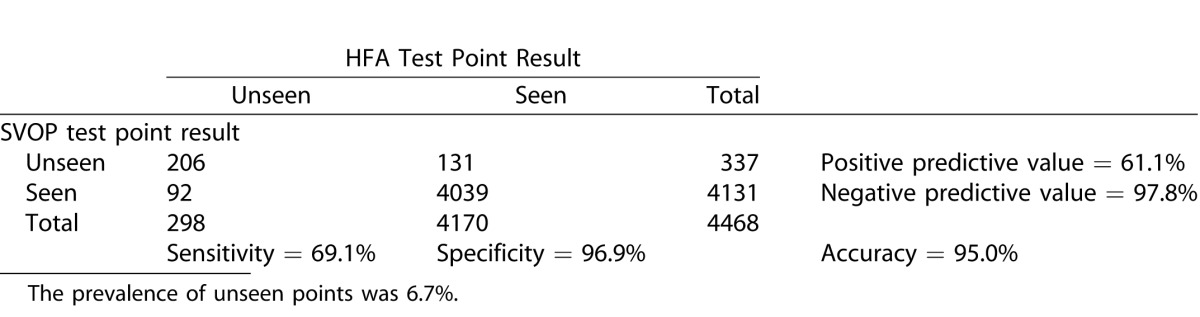
Contingency Table Comparing SVOP Test Point Results with HFA Test Point Results in all Groups

### Repeatability of SVOP and HFA

Tables 11 and 12 detail the Cohen's kappa measurement of agreement values for repeated SVOP and HFA test results (normal or abnormal) and repeated test point results (seen or unseen), respectively.

**Table 11:**
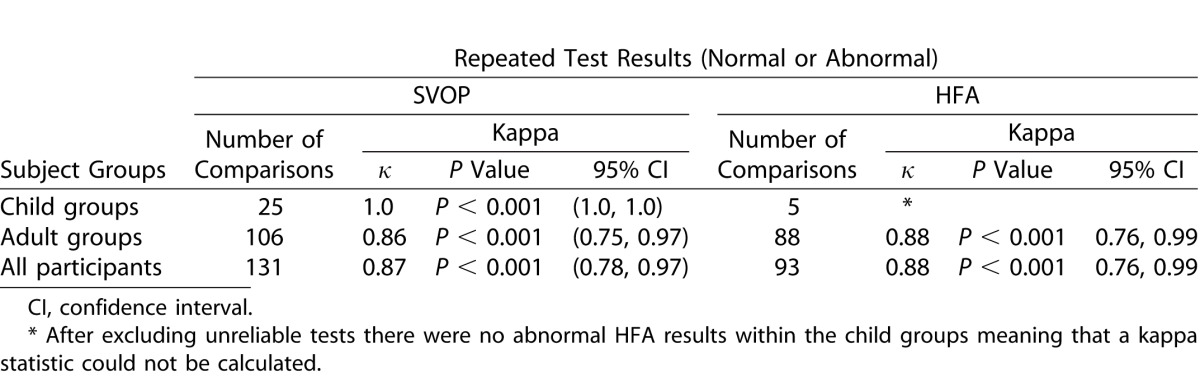
Kappa Statistic, κ, for Analysis of Repeatability of SVOP and HFA Test Results (Normal or Abnormal)

**Table 12:**
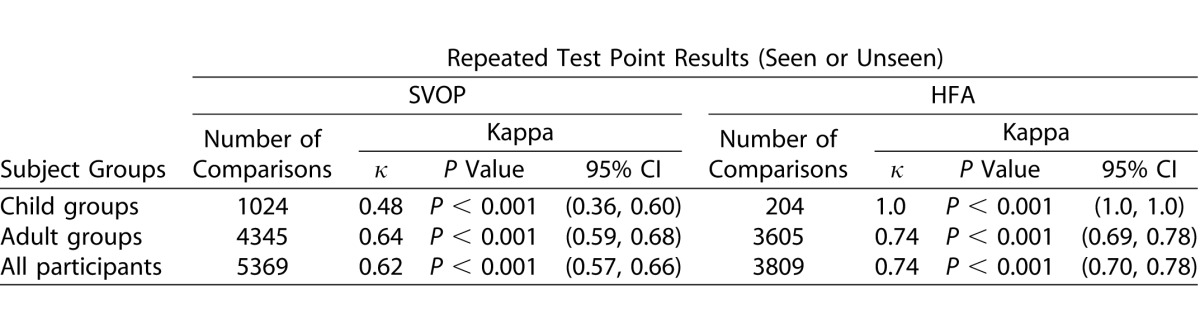
Kappa Statistic, κ, for Analysis of Repeatability of SVOP and HFA Test Point Results (Seen or Unseen)

### Test Times

**Figure 4:**
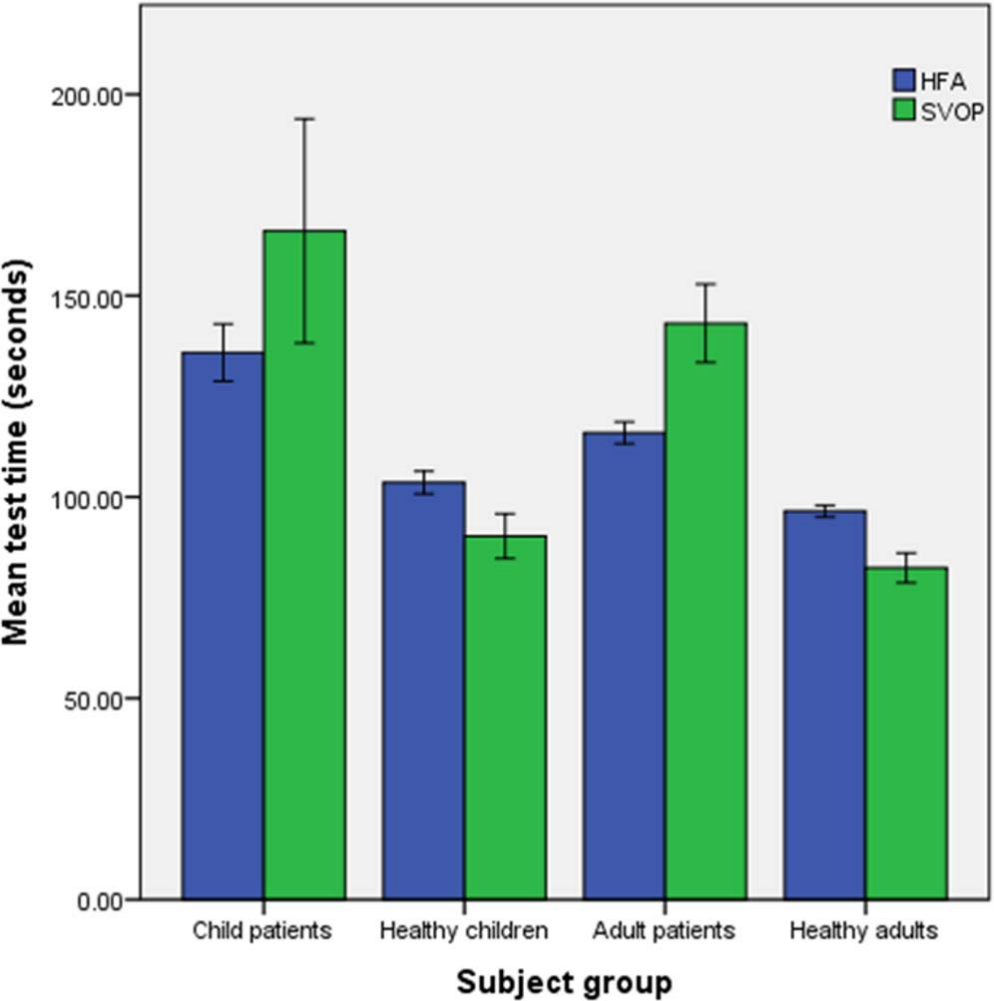
Comparison of test times between SVOP and HFA tests for each of the subject groups. Data shown is the mean (±1 standard error of the mean) of all completed tests from the first test session.

The test times of all SVOP and HFA tests were recorded. Figure 4 shows the test times of completed SVOP and HFA tests for each subject group. The HFA test was on average significantly quicker (*n* = 54, mean = 116.2 seconds, SD = 20.6 seconds) than SVOP (*n* = 54, mean = 143.1 seconds, SD = 71.4 seconds) within the adult patient group (Z = −2.708, *P* < 0.01). In the healthy adult group, SVOP tests were on average quicker (*n* = 58, mean = 82.4 seconds, SD = 2801 seconds) compared with the HFA tests (*n* = 58, mean = 96.5 seconds, SD = 11.6); (Z = −4.107, *P* < 0.001). Also, in the healthy child group the SVOP tests (*n* = 23, mean = 90.3 seconds, SD = 26.6 seconds) were significantly faster than the HFA tests (*n* = 23, mean = 103.0 seconds, SD = 13.8 seconds); (Z = −2.099, *P* < 0.05).

## Discussion

The full test protocol was completed by 82% of the subjects participating in this study. The majority of those with an incomplete protocol were children and adult patients. Of healthy controls, 87% completed the full protocol. In the adult patient group the main reason for incomplete protocol was difficulties in obtaining accurate eye tracking data. The eye tracker used in this SVOP system was purchased off-the-shelf and was designed for a normal population. Our data supports this as no eye tracking problems were noted in the healthy groups in this study. Poor eye tracking can occur when the quality of the image of the pupil margin and corneal reflex is impaired due to factors such as: (1) dry eye and reduced ocular surface integrity, (2) spectacles, (3) irregular pupil shape, (4) strabismus, (5) nystagmus, and (6) eye makeup.^37^ Many of these potential reasons are more likely to appear in an ophthalmology patient population. The eye tracker used in this study is now no longer produced by the manufacturer. Newer models have introduced proprietary developments to improve eye tracking, however the detail of these improvements is not known and further testing on patient groups, as well as collaboration with eye tracking manufacturers, is required in order to better understand these issues. Despite this limitation, the majority of the cohort had good quality eye tracking data and complete test protocols.

In the child patient group, 59% of first session SVOP tests were at least 95% complete, and in healthy children 96% of first SVOP tests were at least 95% complete. This compares favorably with a study performed by Tailor et al.,^32^ in which only 12.5% of child patients completed a 40-point SVOP test. The children in the Tailor et al.^32^ study had quite severe neurodisability. The sensory and motor reflex loop required to generate an accurate saccadic response to a peripheral stimulus is complex, and involves a variety of cortical and subcortical pathways. It is perhaps not surprising that the performance of SVOP will be less satisfactory in children with widespread abnormalities of the central nervous system.

Many of the HFA tests performed by children were rejected due to high rates of false-positive responses and fixation losses. These problems are frequently noted when performing static SAP with children. In this study, the children were naïve users of both the HFA and SVOP tests and no training was provided for either test. In future studies, it would be useful to give a child more time to practice the HFA test to enable a higher rate of reliable results, which can be used in the analysis. These HFA test reliability data demonstrate the inherent problems children have when performing SAP. SVOP can overcome some of these issues. For example, if an individual has poor fixation and is trying to scan and search for the next test stimulus, the SVOP test will not proceed until fixation is maintained on the fixation stimulus. In this way, the test is unlikely to have any fixation losses and fixation itself controls the test. However, a problem with this method is that the test can take longer to perform if the subject has inaccurate fixation or is prone to scanning and searching.

While SVOP was originally designed for children, the aim of this study was to assess the accuracy and reproducibility of SVOP as a visual field assessment technique generally. This required the use of equivalent, accurate HFA tests for comparison. Due to the exclusion of unreliable HFA tests, the analysis performed on the child groups was not robust and due to the iterative nature of the technology it is not possible to add more children to this study. In future SVOP studies involving children an increased number of participants is required, and alternative forms of visual field test comparison are needed. Tailor et al.^32^ found there was 50% clinical agreement between SVOP and confrontation fields in young children, and 64.7% clinical agreement between SVOP and Goldman visual fields in older children. In our study, sensitivity and specificity for SVOP compared with HFA were 91% and 89% in adults. Testing children remains challenging and work is ongoing to improve the decision algorithms used in SVOP while at the same time eye tracking technology also continues to advance.

Using the HFA visual fields as a reference standard to assess its accuracy, SVOP had an overall (across all subject groups) sensitivity and specificity of 91% and 89%, respectively, when assessing the entire visual field as abnormal or normal. A limitation of this analysis method is that a visual field could be abnormal in one particular area with one test and in an entirely different area of the visual field with the other test but the outcome of both would be abnormal. In light of this, a more specific comparison was made by comparing all individual test points. By analyzing the data in this way SVOP had an overall sensitivity and specificity of 69% and 97%, respectively. Overall the reproducibility of the HFA and SVOP tests was categorized as ‘excellent' (Cohen's kappa of 0.80 and 0.83, respectively) when assessing the full visual field result as normal or abnormal. When assessing the individual visual field points, the reproducibility of SVOP and HFA were both categorized as ‘substantial' but with HFA scoring higher than SVOP (Cohen's kappa of 0.74 and 0.62, respectively).

The SVOP tests were significantly faster than the HFA tests in the healthy groups but slower in the patient groups. One reason for this is retesting of unseen points. Both the SVOP and the HFA suprathreshold tests retest points if they are initially unseen, however it is not known if the HFA retests all initially unseen points or if it uses a more sophisticated algorithm to determine if points need to be retested. Additionally, the SVOP test has a static time-period during which it waits for an eye movement response to visual field stimuli before deciding that a stimulus is unseen. The HFA uses a dynamic time-period, which reduces as the test progresses if the subject has a reliable response time. These practices could be added to SVOP in order to improve test times further, however the data from the normal subjects demonstrates that perimetry using eye movements has the potential to be faster than that which uses a button press.

One limitation of the test time data analyzed is that it does not take into account the time taken to set up testing for either type of test. Each test requires the patient be initially positioned and instructed. Additionally SVOP requires an eye tracking calibration sequence (lasting approximately 20 seconds) and HFA tests also requires an eye tracking calibration if its fixation monitoring functionality is used. These times were not analyzed in this study.

Using equivalent HFA tests as a reference standard, suprathreshold SVOP was an accurate visual field test in this group of adults. Further studies using alternative reference standards for children are required in order to assess SVOP in child patient groups and this is crucial as the technology matures. This study has shown that it is possible to use saccades as a response mechanism in perimetry. This has also been demonstrated by other groups using eye movement perimetry (EMP).^38^ In addition, using saccades as the response mechanism may provide an additional measure for assessing glaucoma due to the increased saccadic reaction times seen in glaucoma patients as compared with healthy controls.^39^ The results of this study provide a benchmark for future iterations of the SVOP technique.
